# Eldecalcitol effectively prevents alveolar bone loss by partially improving Th17/Treg cell balance in diabetes-associated periodontitis

**DOI:** 10.3389/fbioe.2023.1070117

**Published:** 2023-02-03

**Authors:** Ruihan Gao, Weidong Zhang, Yujun Jiang, Junzhe Zhai, Jian Yu, Hongrui Liu, Minqi Li

**Affiliations:** ^1^ Department of Bone Metabolism, School and Hospital of Stomatology, Cheeloo College of Medicine, Shandong University & Shandong Key Laboratory of Oral Tissue Regeneration & Shandong Engineering Laboratory for Dental Materials and Oral Tissue Regeneration & Shandong Provincial Clinical Research Center for Oral Diseases, Jinan, China; ^2^ Center of Osteoporosis and Bone Mineral Research, Shandong University, Jinan, China; ^3^ Department of Radiology, School and Hospital of Stomatology, Cheeloo College of Medicine, Shandong University & Shandong Key Laboratory of Oral Tissue Regeneration & Shandong Engineering Laboratory for Dental Materials and Oral Tissue Regeneration, Jinan, China

**Keywords:** diabetes-associated periodontitis, periodontitis, Th17/Treg balance, eldecalcitol, STAT3/STAT5 signaling

## Abstract

**Background:** Diabetes-associated periodontitis (DPD) is an inflammatory and destructive disease of periodontal tissues in the diabetic population. The disease is manifested as more severe periodontal destruction and is more difficult to treat when compared with periodontitis (PD). Eldecalcitol (ELD) is a novel active vitamin D3 analog; however, little clinical evidence is available on its role on improving PD and DPD, and its specific mechanisms remain unclear. In this study, we evaluated the preventative effects of ELD toward PD and DPD and explored its underlying molecular mechanisms.

**Methods:** Experimental PD and DPD mouse models were established by ligation combined with lipopolysaccharide (LPS) from *Porphyromonas gingivalis* injection in C57BL/6J and C57BLKS/J Iar- + Leprdb/+Leprdb (db/db) mice, respectively. Simultaneously, ELD (0.25 μg/kg) was orally administered to mice *via* an intragastric method. Micro-computed tomography (CT), hematoxylin-eosin (HE) staining, immunohistochemistry (IHC), and tartrate-resistant acid phosphatase (TRAP) staining were used to evaluate alveolar bone alterations *in vivo*. Flow cytometry, immunofluorescence, and real-time polymerase chain reaction (qRT-PCR) were also used to examine gene expression and probe systemic and local changes in Treg and Th17 cell numbers. Additionally, western blotting and immunofluorescence staining were used to examine changes in STAT3/STAT5 signaling.

**Results:** Micro-CT and HE staining showed that the DPD group had higher alveolar bone loss when compared with the PD group. After applying ELD, alveolar bone loss decreased significantly in both PD and DPD groups, and particularly evident in the DPD group. IHC and TRAP staining also showed that ELD promoted osteoblast activity while inhibiting the number of osteoclasts, and after ELD treatment, the receptor activator of nuclear factor-κB ligand (RANKL) to osteoprotegerin (OPG) ratio decreased. More importantly, this decreasing trend was more obvious in the DPD group. Flow cytometry and qRT-PCR also showed that the systemic Th17/Treg imbalance in PD and DPD groups was partially resolved when animals were supplemented with ELD, while immunofluorescence staining and qRT-PCR data showed the Th17/Treg imbalance was partially resolved in the alveolar bone of both ELD supplemented groups. Western blotting and immunofluorescence staining showed increased p-STAT5 and decreased p-STAT3 levels after ELD application.

**Conclusion:** ELD exerted preventative effects toward PD and DPD by partially rectifying Th17/Treg cell imbalance *via* STAT3/STAT5 signaling. More importantly, given the severity of DPD, we found ELD was more advantageous in preventing DPD.

## 1 Introduction

Periodontitis (PD) is a chronic infectious disease in teeth-supporting tissue and is characterized by microbially‐associated and host‐mediated inflammation that causes periodontal attachment loss (Tonetti et al., 2018). Thanks to greater insights from periodontal medicine research, correlations between periodontitis and systemic diseases have been proposed (Cecoro et al., 2020). Several studies have confirmed a mutual relationship between periodontitis and diabetes: diabetes is an important risk factor of periodontitis, and persistent periodontal infection can increase difficulties controlling blood glucose (Barutta et al., 2022). A recent meta-analysis reported that the prevalence of periodontitis in diabetic patients was twice that of non-diabetic patients (Zheng et al., 2021). Furthermore, studies have listed periodontitis as the sixth complication of diabetes, known as diabetes-associated periodontitis (DPD) (Löe, 1993). When compared with PD, DPD has more complex systemic factors, increased rapid alveolar bone resorption rates, greater reduced periodontal tissue repair capabilities, and less satisfactory clinical treatment outcomes (Barutta et al., 2022). Therefore, it is vital to explore DPD pathogenesis and identify preventative measures.

In recent years, DPD pathogenesis has attracted considerable research attention, with multiple reported mechanisms, including microbial factors, immune homeostasis imbalance, and oxidative stress (Polak and Shapira, 2018). It is accepted that the initiating factors for PD are dental plaque biofilms (Valm, 2019). Furthermore, accumulating evidence now suggests that the cause of accelerated periodontal destruction in diabetes mellitus is not solely related to changes in microbial pathogenicity but more to imbalanced host immune responses (Bosshardt, 2018). Moreover, it was reported that PD and diabetes shared common immune characteristics, e.g., chronic inflammation and imbalanced immune homeostasis (Zou et al., 2022). Consequently, these factors are critical for the mutual promotion of diabetes and PD and exploring imbalanced immune homeostasis is important to fully characterize DPD pathogenesis (Luong et al., 2021).

CD4^+^ T helper cell (CD4^+^ T cell)-mediated cellular immune responses have important roles in immune homeostasis (Zhu and Zhu, 2020). CD4^+^ T cells comprise significant cell subsets which respond to different immune environments, including Th1, Th2, Th17, and Treg (T regulatory), and Tfh (follicular T helper) cells. In recent years, Th17 and Treg cell functions have aroused particular concerns (Lee, 2018). Th17 cells exert pro-inflammatory effects by secreting interleukin-17 (IL-17), which has multiple cell sources, such as Th17 cells (main sources), γδT cells, LTi cells, epithelial cells and so on (Gu et al., 2013). Alternatively, Treg cells have important roles in immunological self-tolerance, and prevent excessive damage by the immune system. Clinical and animal model studies have indicated that Th17/Treg cell imbalance, i.e., increased Th17 cell and decreased Treg cell proportions, is a common immune mechanism in periodontitis and diabetes (Kou et al., 2021; Li et al., 2022). More importantly, this imbalance has a vital role in the reciprocal relationship between diabetes and periodontitis; periodontitis exacerbates insulin resistance and blood glucose control by imbalancing Th17/Treg cell levels (Tao et al., 2019). Conversely, diabetes-mediated Th17/Treg cell imbalance massively activates and releases inflammatory factors, such as IL-17, thereby destroying periodontal tissue (Huang et al., 2020a). Hence, correcting the imbalance of Th17/Treg may be an important strategy preventing and treating DPD.

The active form of vitamin D is metabolized by the kidney and liver and has classical biological functions in regulating calcium and phosphorus metabolism and improving osteoporosis (Ringe, 2020). In recent years, active vitamin D function in regulating the immune system has attracted much research attention (Ribeiro et al., 2022). It was reported that active vitamin D reduced CD4^+^ T cell differentiation into Th17 cell subsets, and altered them to Treg cells, thereby providing protective effects toward Th17/Treg cell imbalance (Ji et al., 2019). Eldecalcitol (ELD) is a novel active vitamin D analog with anti-osteoporosis properties (Liu et al., 2022). Since ELD was used in clinical treatment of osteoporosis in 2011, it has achieved great clinical evaluation, which can effectively increase bone mineral density (BMD) and promote bone mineralization (Matsumoto et al., 2011). Compared with the original active vitamin D, its half-life in the blood is longer, so that the bioavailability is greatly improved; in addition, it makes up for the defect of calcium-phosphate imbalance caused by the original active vitamin D, and achieves the optimization of medicinal property (Saito et al., 2017). Studies have also shown that ELD can effectively regulate calcium homeostasis (Jiang et al., 2019), inhibit bone resorption (Han et al., 2017), and induce unique “mini-modeling” bone formation (de Freitas et al., 2011). Our group has systematically studied the effects and mechanisms underpinning ELD, and observed good hypoglycemic effects (Lu et al., 2022). Additionally, we confirmed ELD protective effects toward gingival fibroblasts, thereby providing a theoretical basis for preventing PD (Huang et al., 2020b). However, the beneficial effects of ELD toward DPD remain largely unreported.

Given the regulatory effects of active vitamin D on Th17/Treg cell imbalance and the aforementioned ELD effects, we explored the effects of ELD on DPD and analyzed its underlying mechanisms to provide a theoretical basis for a better understanding of DPD pathogenesis. Also, using ELD in such situations provides new ideas for preventing and treating DPD.

## 2 Materials and methods

### 2.1 Animal and tissue preparation

Eighteen male C57BL/6J mice, weighing 25–30 g and aged 8 weeks, were purchased from the animal center of Shandong University. Additionally, twelve male C57BLKS/J Iar- + Lepr^db^/+Lepr^db^ mice (db/db), weighing 33–45 g and aged 8 weeks, were purchased from SPF Biotechnology [SPF (Beijing) Biotechnology Co., Ltd. China] as a diabetic mouse model. Mice were fed under standard laboratory conditions and subjected to light and dark condition for 12 h at 20°C. The study protocol was approved by the Experimental Animal Medicine Ethics Committee of Shandong University School of Stomatology (No. 20211211).

C57BL/6J mice were randomly assigned to three groups (*n* = 6): control (Control), experimental periodontitis (PD), and experimental periodontitis fed ELD (PD + ELD). Additionally, db/db mice were randomly divided into two groups (n = 6): experimental periodontitis (DPD) and experimental periodontitis fed ELD (DPD + ELD). The experimental periodontitis model was established according to a previous method (Teramachi et al., 2017). After mice were anesthetized by the intraperitoneal injection of 1% pentobarbital (50 mg/1000 g body weight), a silk thread (No. 5-0) ligation was performed bilaterally on the maxillary second molar cervical area. Every other day for 2 weeks, 1.0 mg/mL lipopolysaccharide (LPS) from *Porphyromonas gingivalis* (InvivoGen, United States) in 10 μL was injected into the gingival tissue near the upper second molar (Li et al., 2020). Mice in the control group were injected with the same volume of phosphate buffer saline (PBS) (pH 7.4) for 2 weeks without ligating the corresponding area. In addition to establishing an experimental periodontitis model, mice in PD + ELD and DPD + ELD groups were orally treated three times a week for 2 weeks with 0.25 μg/kg ELD (Chugai Pharmaceutical Co., Ltd. Japan) (Hirota et al., 2018). During drug intervention procedures, mice were deprived of water and food for 8 h every 3 days, the tail vein punctured, and fasting blood glucose levels measured using a blood glucose meter (Yuwell Medical Equipment Co., Ltd. China). After 2 weeks, animals were humanely euthanized *via* anesthetic overdose (intraperitoneal injection of 1% pentobarbital at 100 mg/1000 g body weight). Spleens were removed under sterile conditions for flow cytometry studies and RNA extraction. Maxilla were dissected and some components stored at −80°C. The remaining samples were immersed in 4% paraformaldehyde for 24 h and analyzed using a micro-computed tomography (CT) system to assess alveolar bone loss. The remaining samples were demineralized in 10% EDTA-2Na at 4°C for 1 month, dehydrated in ethanol, embedded in paraffin using standard procedures, and prepared as 5-μm sections for histological analyses.

### 2.2 Micro-CT assessments

Three maxilla samples from each group were randomly selected for scanning (SCANCO Medical AG, Switzerland). Voltage = 70 kV, current = 200 μA, and layer thickness = 10 µM. Scanning was performed from the proximal edge of the first molar to the distal edge of the third molar along the long maxilla axis, and the bone tissue of the proximal and distal alveolar bone of the maxillary second molar was used to analyze alveolar bone alterations. All samples were reconstructed and analyzed using a micro-CT analysis system (SCANCO Medical AG). The distance (mm) from the lingual alveolar bone of the maxillary second molar to the cementoenamel junction (CEJ) was measured using Image Pro Plus 6.2 software (Media Cybernetics, Inc. United States).

### 2.3 Splenic lymphocyte isolation and flow cytometry

Spleens were removed under aseptic conditions and dissected on a 70-μm cell sieve. Splenic cell suspensions were collected, and after isolating mononuclear cells using mouse Lymphocyte Isolation Solution (Dakewe Biotech Co., Ltd. China), single cells were suspended in RPMI 1640 medium (Biological Industries, Göttingen, Germany) plus 10% fetal bovine serum (FBS). Cells were stained with fluorescein isothiocyanate-conjugated anti-CD4 antibody (100,406, BioLegend, Inc. CA, United States) for 30 min on ice. After permeabilization and fixation in eBioscience™ FOXP3/transcription factor staining buffer set (Thermo Fisher Scientific, Waltham, MA, United States), cells were incubated with phycoerythrin-conjugated anti- FOXP3 antibody (563,101, BD Biosciences, United States) and allophycocyanin-conjugated anti-IL-17A antibody (506,916, BioLegend) for 1 h at 4°C. Finally, 0.2 mL PBS was added and cells resuspended. Labeled cells were detected by flow cytometry (BD Accuri C6 Plus, United States), and Treg (CD4^+^ FOXP3^+^) and Th17 (CD4^+^ IL-17A^+^) cell percentages determined. Data were analyzed using FlowJo™ v10.8 Software (BD Life Sciences, United States). All experiments were independently performed three times.

### 2.4 Hematoxylin-eosin (HE) staining

Alveolar bone sections were dewaxed and rehydrated for HE staining. Sections were immersed in hematoxylin for 15 min, washed twice in distilled water, and stained with eosin for 7 min. After dehydration and mounting, stained sections were observed under optical microscopy (BX-53; Olympus Corp. Japan), and digital images captured at original magnifications of ×100. The distance from the alveolar bone of the maxillary second molar to the CEJ was measured using Image Pro Plus 6.2 software (Media Cybernetics). Specifically, three tissue sections were selected to quantitative mean histological values.

### 2.5 Immunohistochemistry (IHC) and tartrate-resistant acid phosphatase (TRAP) staining

Sections underwent IHC staining to examine alkaline phosphatase (ALP), receptor activator of nuclear factor-κB ligand (RANKL), and osteoprotegerin (OPG) expression. After dewaxing and rehydration, tissue sections were immersed in PBS plus 0.3% hydrogen peroxide (H_2_O_2_) for 30 min to inhibit endogenous peroxidase, followed by blocking in 1% bovine serum albumin (BSA) in PBS (1% BSA-PBS) for 20 min to prevent non-specific staining. Sections were then incubated overnight at 4°C with: (Tonetti et al., 2018) anti-ALP antibody (1:50; ab65834, Abcam, United Kingdom), (Cecoro et al., 2020) anti-RANKL antibody (1:75; ab216484, Abcam), or (Barutta et al., 2022) anti-OPG antibody (1:100; ab9986, Abcam). After rinsing in PBS, sections were incubated with goat anti-rabbit IgG H&L antibody (1:200, ab6721, Abcam) for 1 h. Immunoreactivity was observed using diaminobenzidine (Sigma-Aldrich, Germany). Sections were washed in double-distilled water and immersed in a TRAP staining solution (Li et al., 2013). All sections were then re-stained in methyl green, sealed, observed under optical microscopy (Olympus Corp), and digital images captured.

For statistical analysis, three sections were selected for quantitative histological measurements, and average values calculated. Furthermore, serial sections were selected and a consistent field recorded for different markers. TRAP-positive osteoclasts were counted at an original magnification of ×200, while at × 400, the mean optical density of ALP, RANKL, and OPG levels were determined in three randomly selected non-overlapping microscopic fields using Image-Pro Plus 6.2 software (Media Cybernetics). Using this approach, regions of interest were manually selected in a color cube.

### 2.6 Immunofluorescence staining

For immunofluorescence staining, paraffin-embedded sections were immersed in 0.3% H_2_O_2_ for 30 min and blocked in 1% BSA-PBS for 20 min. Double immunofluorescence staining was performed on sections to localize Treg cells, Th17 cells, p-STAT3^+^IL-17A^+^ cells and p-STAT5^+^FOXP3^+^ cells. Sections were incubated overnight at 4°C with the following mixed antibodies: (Tonetti et al., 2018) anti-IL-17A antibody (1:100; ab189377, Abcam) and anti-CD4 antibody (1:300; ab183685, Abcam), (Cecoro et al., 2020) anti-FOXP3 antibody (1:100; ab253297, Abcam) and anti-CD4 antibody (1:300; ab183685, Abcam), (Barutta et al., 2022) anti-IL-17A antibody (1:100; ab189377, Abcam) and anti-p-STAT3 (1:100; ab76315, Abcam), (Zheng et al., 2021) anti-FOXP3 antibody (1:100; ab253297, Abcam) and anti-p-STAT5 antibodies (1:100, AF3304, Affinity Biosciences, United States). Sections were incubated with mixed green fluorescent goat anti-rabbit IgG H&L (Alexa Fluor 647) (1:200; ab150081, Abcam) and red fluorescent goat anti-mouse IgG H&L (Alexa Fluor 488) (1:200; ab150119, Abcam) antibodies at room temperature in the dark for 1 h. After washing three times in PBS, samples were nuclear stained with 4′,6-diamidino-2-phenylindole (ab104139, Abcam) for 5 min. Images were obtained under inverted fluorescence microscopy (DMi8 S; Leica, Germany) and the periodontal membrane near the root of the maxillary second molar was selected as the area of interest. All cells were counted at × 400 magnification of the original magnification in immunofluorescence images.

### 2.7 Real-time polymerase chain reaction (qRT-PCR) analysis

To extract RNA from bone, liquid nitrogen was continuously and rapidly added to grind down alveolar bone until a powder was generated. Also, spleens were removed under sterile conditions and lymphocytes isolated to extract RNA. Total RNA was extracted from bone and cell samples using Trizol reagent (AG21102, Accurate Biotechnology Co., Ltd. China). cDNAs were synthesized using the Evo M-MLV RT Reverse Transcription kit II (AG11711, Accurate Biotechnology Co., Ltd.) according to manufacturer’s instructions. Using qRT-PCR instrumentation (LightCycler^®^ 96 SW 1.1; Roche Ltd., Switzerland), qRT-PCR was performed using the SYBR Green Pro Taq HS premixed qRT-PCR kit (AG11701, Accurate Biotechnology). Treg cell-related factors (transforming Growth Factor-β (TGF-β) and FOXP3) and Th17 cell-related factors (retinoic acid-related orphan receptor γ (ROR-γt) and IL-17A) were analyzed. Glyceraldehyde 3-phosphate dehydrogenase (GAPDH) was used as an internal control, and results were presented as relative gene expression. Fold-change in gene expression was calculated relative to controls using the 2^-∆∆Cq^ method in GraphPad Prism software (GraphPad Inc. United States). Primer sequences are shown (Table 1).

**TABLE 1:** Specific primers for control and target genes.

Gene	Forward	Reverse
FOXP3	5′-AGT​GCC​TGT​GTC​CTC​AAT​GGT​C-3′	5′-GGT​GAA​GGT​CGG​TGT​GAA​CG-3
TGF-β	5′-TAC​GGC​AGT​GGC​TGA​ACC​AA-3′	5′-AGG​GCC​AGC​ATA​GGT​GCA​AG-3′
IL-17A	5′-TCA​GAC​TAC​CTC​AAC​CGT​TCC​A-3′	5′-CGG​TTC​ATG​TCA​TGG​ATG​GTG-3′
ROR-γt	5′-GCT​CCA​TAT​TTG​ACT​TTT​CCC​ACT-3′	5′-GAT​GTT​CCA​CTC​TCC​TCT​TCT​CTT​G-3′
GAPDH	5′-CTCGCTCCTGGAAGA TGGTG-3	5′-GGTGAAGGTCGGTGTGAACG-3

https://www.jianguoyun.com/p/DUv82SwQ-ZWHCxjUkt8EIAA.

### 2.8 Western blotting

As described, alveolar bone was ground to a powder and RIPA lysis buffer (Lot 02408/60412, CwBio Biotechnology Co., Ltd. China) used to extract proteins. Protein concentrations were determined using a bicinchoninic acid protein assay detection kit (P0012S, BeyoTime Biotechnology, China). Samples were mixed with a 1/4 volume of 5× sodium dodecyl sulfate loading buffer and heated to 95°C for 5 min. Proteins then underwent sodium dodecyl sulfate-polyacrylamide gel electrophoresis, were transferred to polyvinylidene fluoride membranes, and blocked in 5% BSA in Tris buffered saline with Tween-20 (TBST) (5% BSA-TBST) at room temperature for 1 h. Membranes were incubated overnight at 4°C with the following: (Tonetti et al., 2018) anti-p-STAT3 (1:1000; ab76315, Abcam), (Cecoro et al., 2020) anti-p-STAT5 antibodies (1:1000, AF3304, Affinity Biosciences), (Barutta et al., 2022) anti- STAT3 (1:1000; ab68153, Abcam), (Zheng et al., 2021) anti- STAT5 (1:1000; ab32043, Abcam) and followed by incubation with a horseradish peroxidase-conjugated goat anti-rabbit IgG antibody (1:1000, ab6721, Abcam) for 1 h. Immunoreactive bands were detected using enhanced chemiluminescence reagent (B500024, Proteintech, United States) and a gel imaging system (Amersham Imager 600; General Electric Company, United States) to capture images. Image-Pro Plus 6.2 software (Media Cybernetics) was used to analyze and quantify grayscale values normalized to GAPDH levels. Experiments were repeated at least three times.

### 2.9 Statistical analysis

Statistical analysis was performed in GraphPad Prism software (GraphPad Inc., abcamLa Jolla, San Diego, CA, United States). Differences between three or more groups were tested using one-way analysis of variance. All values were expressed as the mean ± standard deviation. *p* < 0.05 was considered statistically significant.

## 3 Results

### 3.1 Aggravated alveolar bone loss in DPD mice when compared with PD mice

**FIGURE 1:**
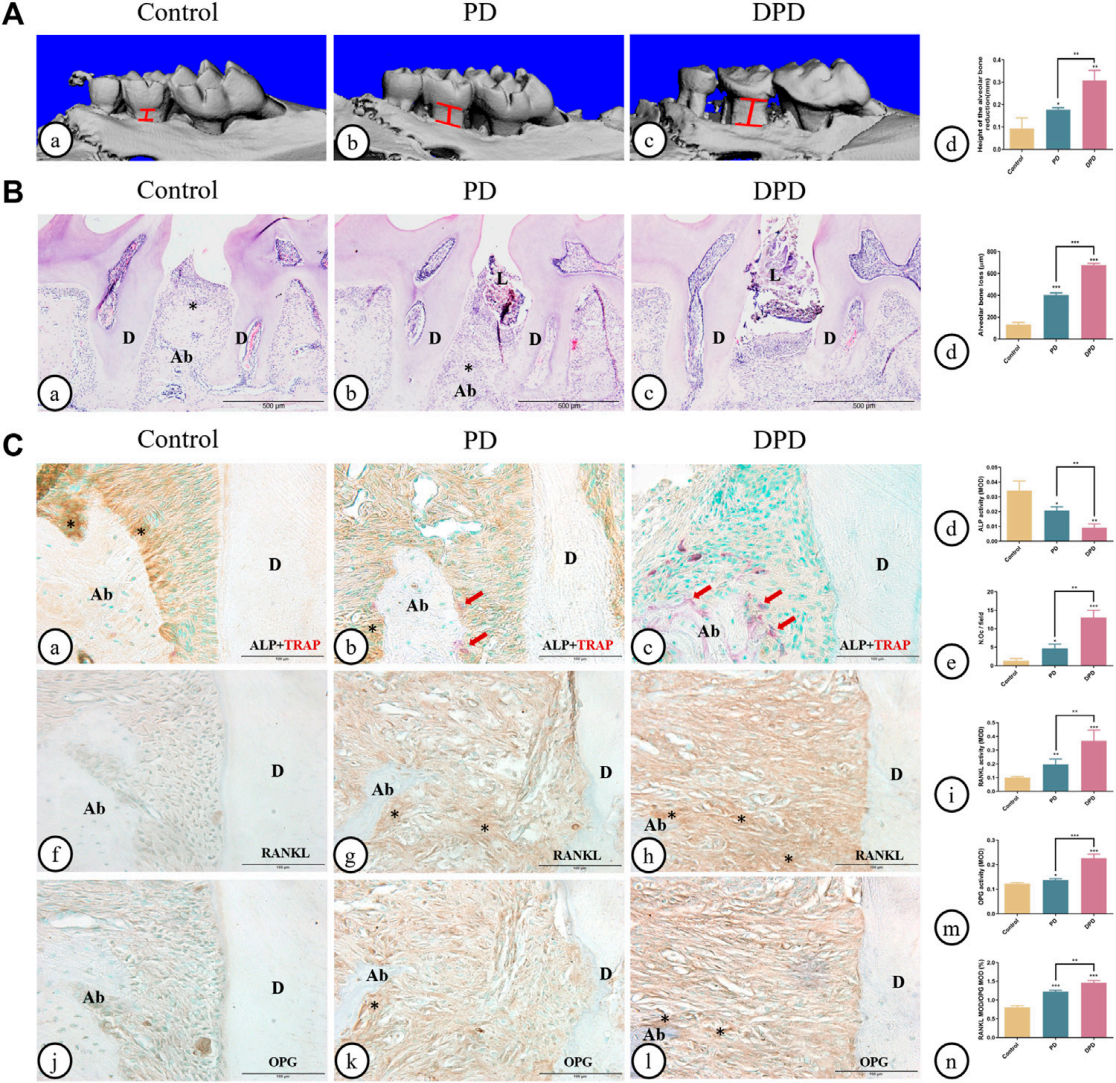
Micro-CT analysis and histological alterations in the maxillary alveolar bone. **(A)** 3D images of micro-CT in the **(A)** control, **(B)** PD and **(C)** DPD groups. (d) Statistical analysis of alveolar bone loss. **(B)** Low magnification of histological images of the alveolar bone in the **(A)** control, **(B)** PD and **(C)** DPD groups. (d) Statistical analysis of alveolar bone loss. “*”, the alveolar ridge crest. **(C)** Double staining for ALP (brown) and TRAP (red) in the alveolar bone of the **(A)** control, **(B)** PD and **(C)** DPD groups. (d) Statistical analyses of ALP positive. (e) TRAP positive osteoclast count. Immunostaining of RANKL in the (f) control, (g) PD and (h) DPD groups. (i) Statistical analysis of the activity of RANKL. Immunostaining of OPG in the (j) control, (k) PD and (l) DPD groups. (m) Statistical analysis of the activity of OPG. (n) The ratio of RANKL/OPG. Positive staining has been marked with arrows or “*” in the figures. Data were shown as mean ± SD (*p < 0.05. **p < 0.01. ***p < 0.001 https://www.frontiersin.org/register. Ab, alveolar bone; D, dentin; L, ligature.

As shown in 3-dimesnional (3D) micro-CT images, in the control group, the alveolar ridge crest (ARC) was near the CEJ. The ARC location was lower in the PD group when compared with the control group, and had a lower location was observed in the DPD group (Figures 1A). Quantitative analyses showed that alveolar bone loss in PD and DPD groups increased when compared with control animals (Figure 1A). In the control group, HE staining showed intact gingival morphology with abundant bone matrix. The periodontal ligament was arranged regularly and alveolar bone around maxillary second molars was intact. However, in PD animals, gingival atrophy and gingival papillae destruction were evident, with only a few bone islands and a deranged periodontal ligament structure distribution. These changes were more pronounced in DPD animals (Figures 1A). Statistical analyses showed that alveolar bone loss increased in PD and DPD groups when compared with the control group, but this loss was more significant in DPD animals (Figure 1B).

The control group exhibited higher ALP-positive osteoblasts on alveolar bone surfaces and periodontal ligaments. In the PD group, ALP-positive osteoblasts decreased and some TRAP-positive osteoclasts were observed around the alveolar bone. When compared with this group, more TRAP-positive osteoclasts and lower ALP-positive osteoblasts were observed in DPD animals (Figures 1A). Statistical analyses also confirmed lower ALP-positive osteoblasts and more TRAP-positive osteoclasts in PD and DPD groups when compared with the control group, while DPD group changes were more significant when compared with the PD group (Figures 1). Relatively weak RANKL expression levels were observed in the control group, but the PD group exhibited increased levels when compared with this group. RANKL expression in the DPD group was the highest of all groups (Figures 1). OPG expression trends were similar (Figures 1J–L). These analyses confirmed our observations, with the RANKL to OPG ratio showing an upward trend (Figures 1). Thus, aggravated alveolar bone loss occurred in DPD when compared with PD.

### 3.2 Higher unbalanced Th17/Treg ratios in DPD when compared with PD mice

**FIGURE 2:**
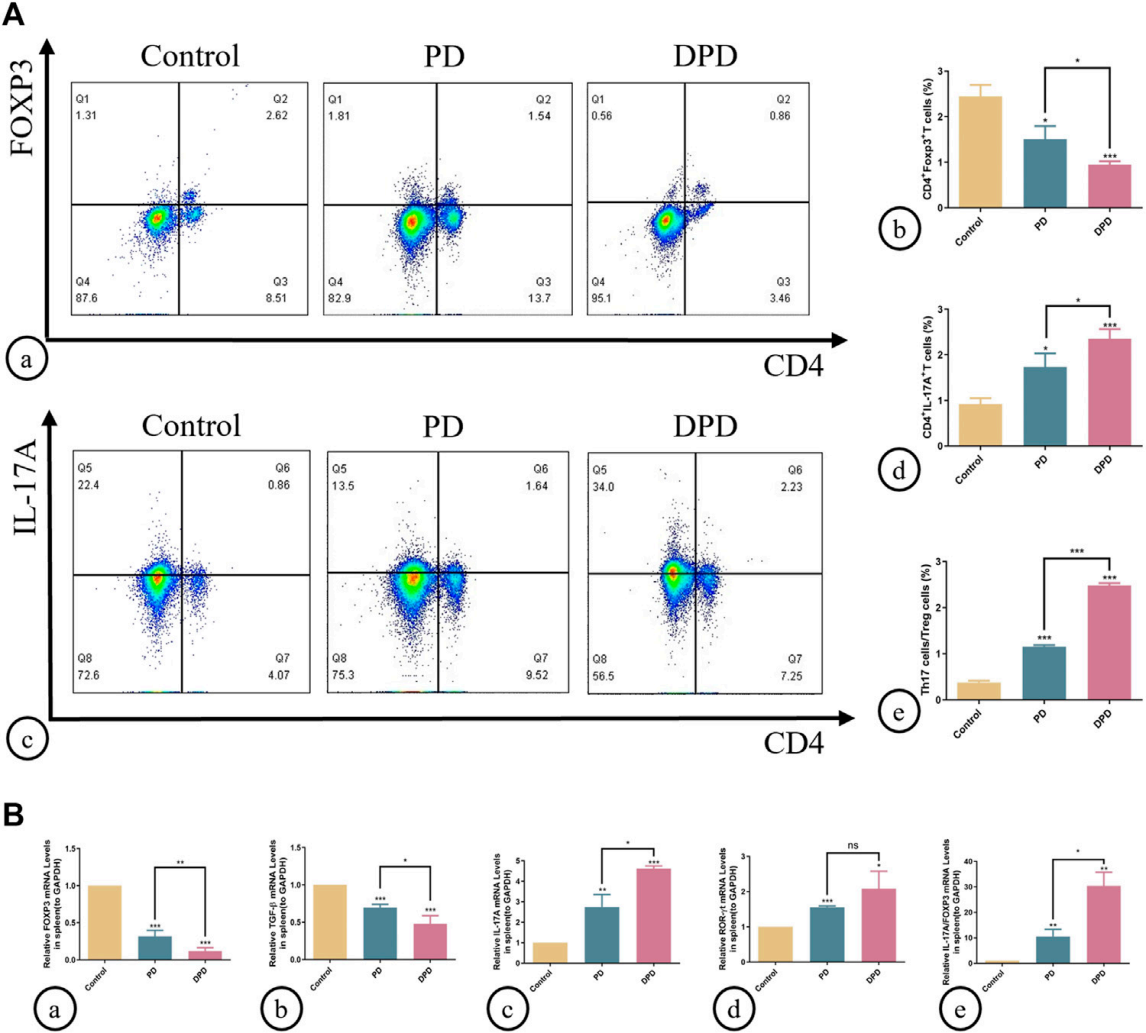
Flow cytometry and qRT-PCR analysis of Th17 and Treg cells in spleen lymphocytes. **(A) (A)** Flow cytometry analysis of CD4^+^ FOXP3^+^ T cells (in the upper right quadrant) in the spleen lymphocytes in the control, PD and DPD groups. (b) Statistical analysis of CD4^+^ FOXP3^+^ T cells in the spleen lymphocytes. (c) Flow cytometry analysis of CD4^+^ IL-17A^+^ T cells (in the upper right quadrant) in the spleen lymphocytes in the control, PD and DPD groups. (d) Statistical analysis of CD4^+^ IL-17A^+^ T cells in the spleen lymphocytes. (e) The ratio of Th17/Treg cells. **(B)** Relative mRNA expression level of **(A)** FOXP3, (b) TGF-β, (c) IL-17A, (d) ROR-ɣt, and (e) the ratio of IL-17A/FOXP3 in the control, PD and DPD groups. Data were shown as mean ± SD (*p < 0.05. **p < 0.01. ***p < 0.001).

As indicated (Figure 2), circulating Treg cells decreased more significantly in DPD mice than in PD mice when compared with control animals (Figures 2A, B). Conversely, circulating Th17 cell percentages increased in PD mice when compared with control animals, whereas levels increased markedly in the DPD group (Figures 2A, C, D). Further evaluations showed that Th17/Treg ratios in PD and DPD groups increased when compared with control animals, particularly in the DPD group, and suggested that Th17/Treg cell proportions in PD and DPD mice were unbalanced when compared with controls. Also, the imbalance in the DPD group was more serious (Figure 2A).

We used qRT-PCR to analyze the expression of Treg (FOXP3 and TGF-β) and Th17 cell-related cytokines (IL-17A and ROR-ɣt) in the spleen. As shown (Figure 2), higher decreases in FOXP3 and TGF-β expression were observed in the DPD group than in the PD group when compared with the control group, while the expression of Th17 cell-related cytokines increased significantly (Figure 2). Further evaluations showed that IL-17A/FOXP3 mRNA expression ratios increased in the DPD group (Figure 2B). Thus, imbalanced Th17/Treg cell were more severe in the DPD group when compared with the PD group.

**FIGURE 3:**
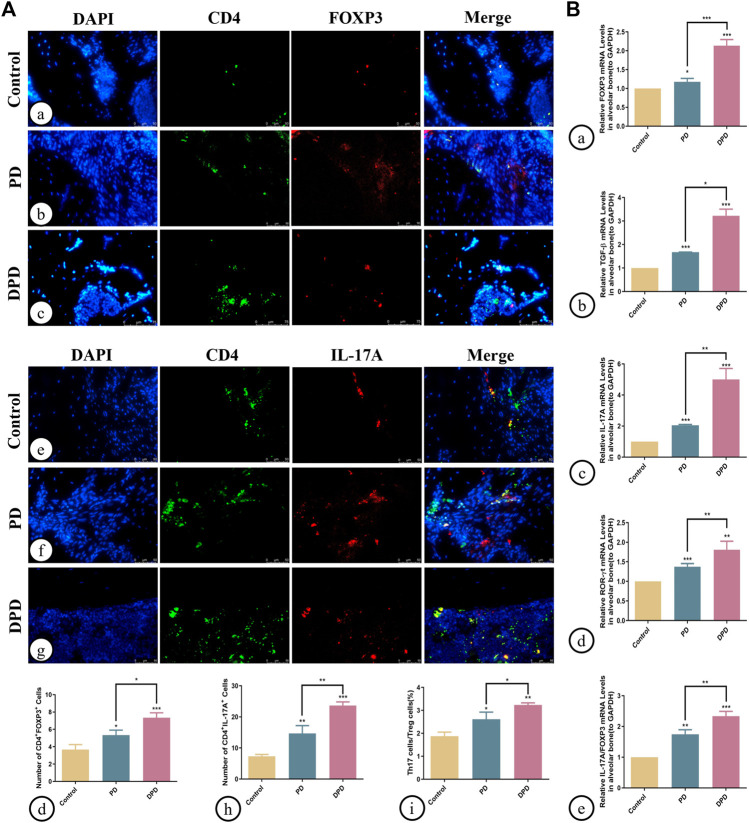
Immunofluorescence and qRT-PCR analysis of Th17 and Treg cells in the maxillary alveolar bone. **(A)** The representative immunofluorescence staining images of CD4^+^ FOXP3^+^ T cells in the (a) control, (b) PD and (c) DPD groups. (d) The statistical analysis of CD4^+^FOXP3^+^ T cell counts. The representative immunofluorescence staining images of CD4^+^ IL-17A^+^ T cells in the (e) control, (f) PD and (g) DPD groups. (h) The statistical analysis of CD4^+^ IL-17A^+^ T cell counts. (i) The ratio of Th17/Treg cells. **(B)** Relative mRNA expression level of (a) FOXP3, (b) TGF-β, (c) IL-17A, (d) ROR-ɣt, and (e) the ratio of IL-17A/FOXP3 in the control, PD and DPD groups. Data were shown as mean ± SD (*p < 0.05. **p < 0.01. ***p < 0.001).

Additionally, we determined Th17/Treg cell proportions in periodontal tissue. Both immunofluorescence staining and statistical analyses showed that Treg cell numbers labeled with both CD4^+^ (green) and FOXP3^+^ (red) fluorescence increased in PD and DPD groups (Figure 3A). Similarly, Th17 cell numbers labeled with both CD4^+^ (green) and IL-17A^+^ (red) fluorescence increased in PD and DPD groups. More strikingly, the DPD group had higher Th17 cell numbers when compared with the PD group (Figure 3A). Further statistical analyses showed that Th17/Treg cell ratios increased in PD and DPD groups when compared with control animals, particularly in the DPD group, suggesting that Th17/Treg cell proportions in alveolar bone was imbalanced in PD and DPD mice, but more severe in DPD animals (Figure 3A).

We also determined Treg and Th17 cell-related cytokine expression in alveolar bone. As shown (Figure 3), increased FOXP3, TGF-β, IL-17A, and ROR-ɣt mRNA expression was observed in PD and DPD groups when compared with control animals, particularly in DPD mice (Figure 3B). Further evaluations showed that IL-17A/FOXP3 mRNA expression ratios increased in PD and DPD groups (Figure 3B). Thus, circulating and local Th17/Treg cell levels were imbalanced in PD and DPD animals, but the imbalance was more severe in DPD mice.

### 3.3 ELD was more advantageous in preventing DPD

**FIGURE 4:**
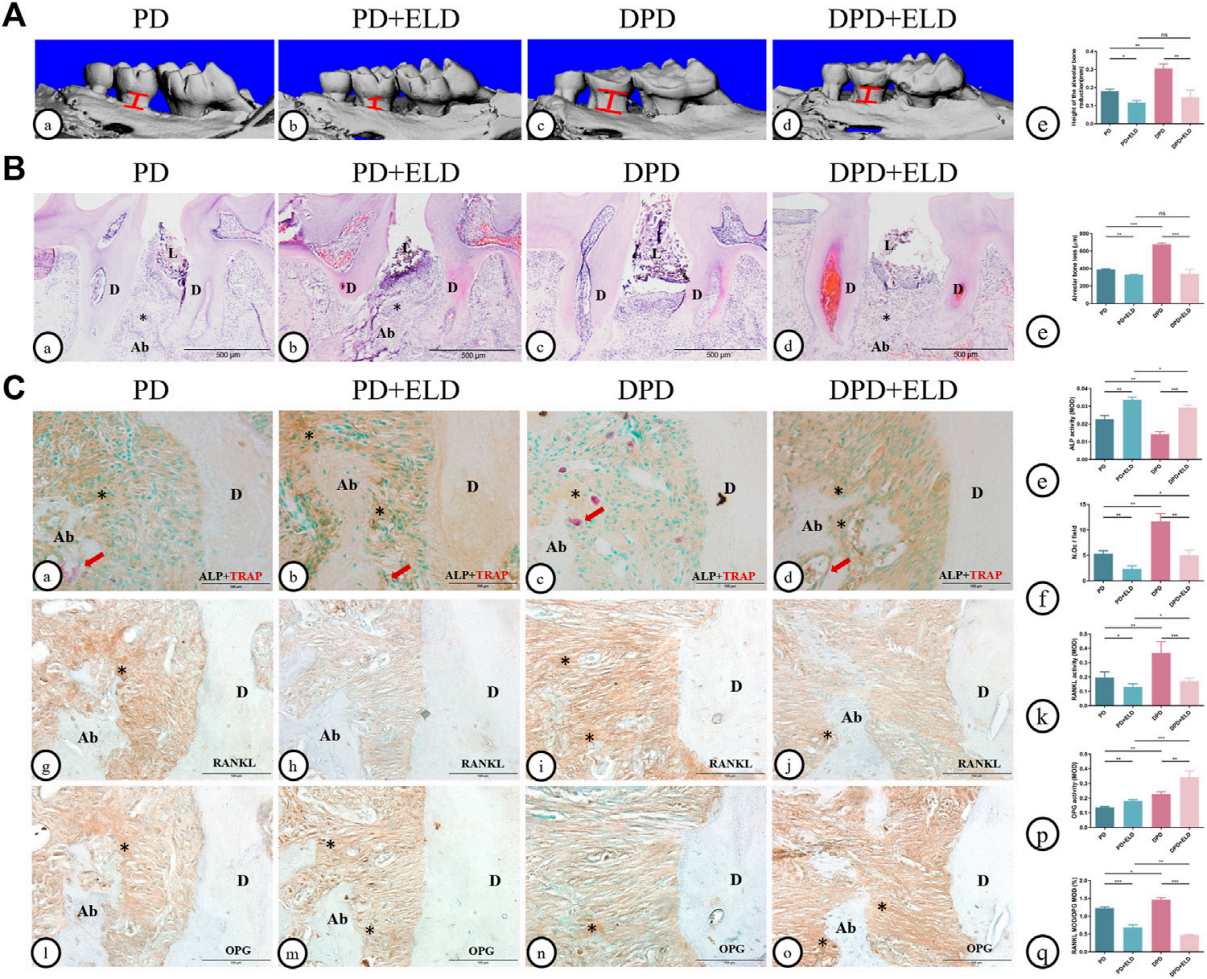
Micro-CT analysis and histological alterations in the maxillary alveolar bone with ELD treatment. **(A)** 3D images of micro-CT in the (a) PD, **(B)** PD + ELD, (c) DPD and (d)DPD + ELD groups. (e) Statistical analysis of the data from micro-CT for alveolar bone loss. **(B)** Low magnification of histological images of the alveolar bone in the (a) PD, **(B)** PD + ELD, (c) DPD and (d) DPD + ELD groups. (e) Statistical analysis of the data from HE staining for alveolar bone loss. “*“, the alveolar ridge crest. **(C)** Double staining for ALP (brown) and TRAP (red) in the alveolar bone of the (a) PD, (b) PD + ELD, (c) DPD and (d) DPD + ELD groups. Statistical analysis of (e) ALP positive and (f) TRAP positive osteoclast counts. Immunostaining of RANKL in the **(G)** PD, (h) PD + ELD, (i) DPD and (j) DPD + ELD groups. (k) Statistical analysis of the activity of RANKL. Immunostaining of OPG in the (l) PD, (m) PD + ELD, (n) DPD and (o) DPD + ELD groups. (*p*) Statistical analysis of the activity of OPG. (q) The ratio of RANKL/OPG. Positive staining has been marked with arrows or “*” in the figures. Data were shown as mean ± SD (*p < 0.05. **p < 0.01. ***p < 0.001). Ab, alveolar bone; D, dentin; L, ligature.

Micro-CT and 3D image data showed that ELD reduced alveolar bone loss in PD and DPD mice. Interestingly, alveolar bone loss height in PD + ELD and DPD + ELD groups was similar. Since alveolar bone resorption was more severe in DPD when compared with PD mice, and ELD restored levels, these findings suggested that ELD was more advantageous in preventing DPD-induced alveolar bone loss when compared with PD (Figure 4A).

HE staining showed that alveolar bone destruction decreased significantly in PD + ELD and DPD + ELD groups when compared with PD and DPD groups, respectively (Figure 4B). Statistical analyses revealed that alveolar bone loss height in PD + ELD and DPD + ELD groups was similar (Figure 4B).

Also, PD + ELD and DPD + ELD groups exhibited increased ALP-positive osteoblasts and fewer TRAP-positive osteoclasts when compared with PD and DPD groups, respectively (Figure 4C). Additionally, RANKL expression had decreased and OPG expression had increased after ELD treatment (Figure 4C). Also, PD + ELD and DPD + ELD groups exhibited decreased RANKL/OPG ratios when compared with PD and DPD groups, respectively and this decreasing trend was more obvious in the DPD group (Figure 4C). These findings suggested that ELD was more advantageous in preventing DPD.

### 3.4 The preventative effect of ELD was achieved by ameliorating Th17/Treg imbalance

**FIGURE 5:**
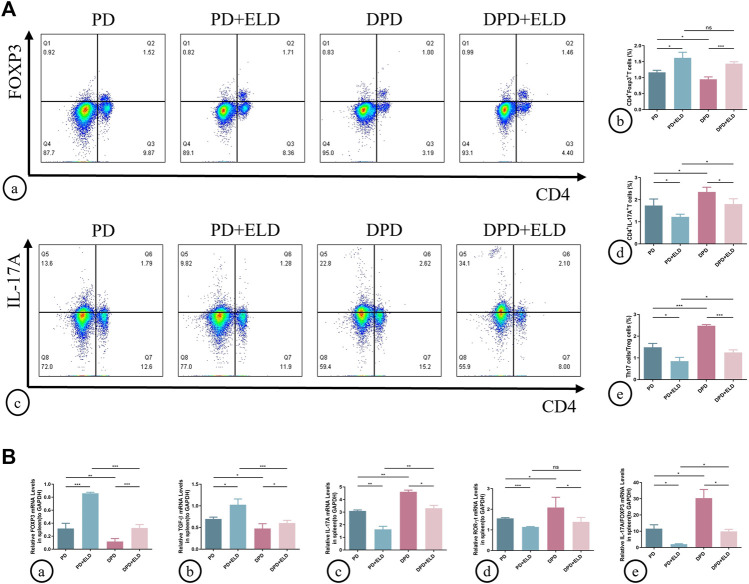
Flow cytometry and qRT-PCR analysis of Th17 and Treg cells with ELD treatment in spleen lymphocytes. **(A)** (a) Flow cytometry analysis of CD4^+^ FOXP3^+^ T cells (in the upper right quadrant) in the spleen lymphocytes in the PD, PD + ELD, DPD and DPD + ELD groups. (b) Statistical analysis of CD4^+^ FOXP3^+^ T cells in the spleen lymphocytes. (c) Flow cytometry analysis of CD4^+^ IL-17A^+^ T cells (in the upper right quadrant) in the spleen lymphocytes in the PD, PD + ELD, DPD and DPD + ELD groups. (d) Statistical analysis of CD4^+^ IL-17A^+^ T cells in the spleen lymphocytes. (e) The ratio of Th17/Treg cells. **(B)** Relative mRNA expression levels of (a) FOXP3, (b) TGF-β, (c) IL-17A, (d) ROR-ɣt and (e) the ratio of IL-17A/FOXP3 in the PD, PD + ELD, DPD and DPD + ELD groups. Data were shown as mean ± SD (*p < 0.05. **p < 0.01. ***p < 0.001).

To determine if ELD prevented alveolar bone loss by preventing Th17/Treg cell imbalance, we examined circulating and local Th17/Treg cell ratios. Flow cytometry showed that spleen Treg cell proportions increased in PD + ELD and DPD + ELD groups when compared with PD and DPD groups, respectively (Figure 5A). But these proportions did not return to control group levels. Moreover, reduced Th17 cell proportions were observed in PD + ELD and DPD + ELD groups. Consistent with Treg cells, Th17 cell proportions in PD + ELD and DPD + ELD groups were still higher when compared with control animals (Figure 5A). Further evaluations showed that Th17/Treg cell ratios decreased in PD + ELD and DPD + ELD groups when compared with PD and DPD groups, suggesting that ELD prevented Th17/Treg cell imbalance. Though Th17/Treg cell ratios in PD + ELD and DPD + ELD groups were still unbalanced, the improvement of Th17/Treg cell imbalance in DPD + ELD group was more obvious than that in PD + ELD group (Figure 5A).

Similarly, Treg and Th17 cell-related cytokine expression was investigated in the spleen. The mRNA expression of FOXP3 and TGF-β increased in PD + ELD and DPD + ELD groups when compared with PD and DPD groups, respectively (Figure 5B). Conversely, the mRNA expression of IL-17A and ROR-ɣt decreased after ELD treatment in PD and DPD group (Figure 5B). Also, IL-17A/FOXP3 mRNA expression ratios decreased in PD + ELD and DPD + ELD groups (Figure 5B). Therefore, ELD exerted preventative effects on Th17/Treg cell imbalance.

**FIGURE 6:**
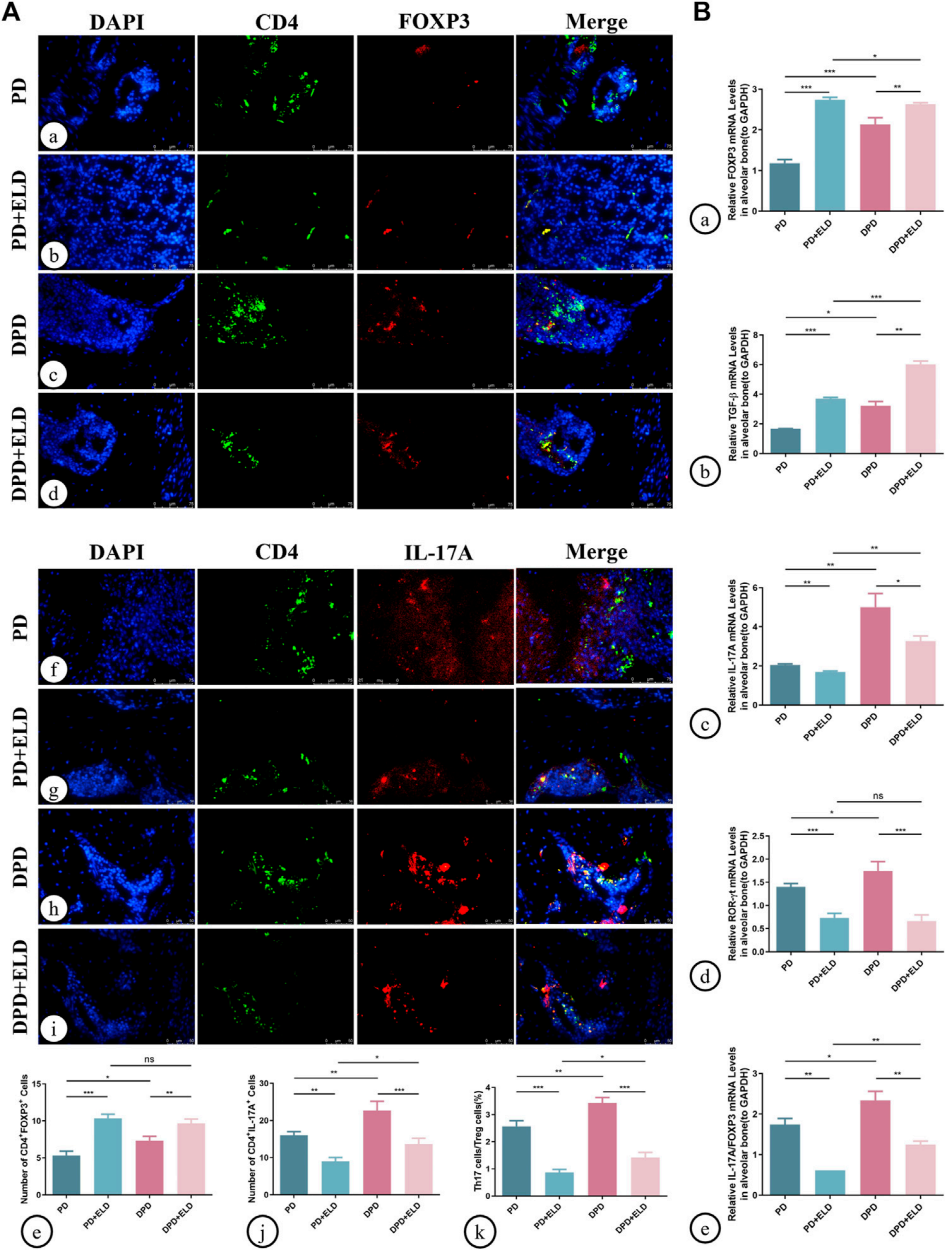
Immunofluorescence and qRT-PCR analysis of Th17 and Treg cells in the maxillary alveolar bone with ELD treatment. **(A)** The representative immunofluorescence staining images of CD4^+^ FOXP3^+^ T cells in the (a) PD, (b) PD + ELD, (c) DPD and (d) DPD + ELD groups. (e) The statistical analysis of CD4^+^ FOXP3^+^ T cell counts. The representative immunofluorescence staining images of CD4^+^ IL-17A^+^ T cells in the (f) PD, (g) PD + ELD, (h) DPD and (i) DPD + ELD groups. (j) The statistical analysis of the number of CD4^+^ IL-17A^+^ T cells. (k) The ratio of Th17/Treg cells. **(B)** Relative mRNA expression levels of **(A)** FOXP3, (b) TGF-β, (c) IL-17A, (d) ROR-ɣt and (e) the ratio of IL-17A/FOXP3 in the PD, PD + ELD, DPD and DPD + ELD groups. Data were shown as mean ± SD (*p < 0.05. **p < 0.01. ***p < 0.001).

Additionally, Th17/Treg cell ratios in alveolar bone were measured by immunofluorescence. As indicated (Figure 6), increased Treg and decreased Th17 cell numbers were identified in PD + ELD and DPD + ELD groups when compared with PD and DPD groups (Figure 6A. Further statistical analyses showed that Th17/Treg cell ratios decreased in PD + ELD and DPD + ELD groups when compared with PD and DPD groups (Figure 6A). Additionally, the detection of Treg and Th17 cell-related cytokines by qRT-PCR also confirmed that ELD have the preventative effect on the imbalance of Th17/Treg (Figure 6B). These findings suggested that ELD has an effective preventive effect on local Th17/Treg cell imbalance.

### 3.5 The preventative effects of ELD on Th17/Treg cells imbalance *via* STAT3/STAT5 signaling

**FIGURE 7:**
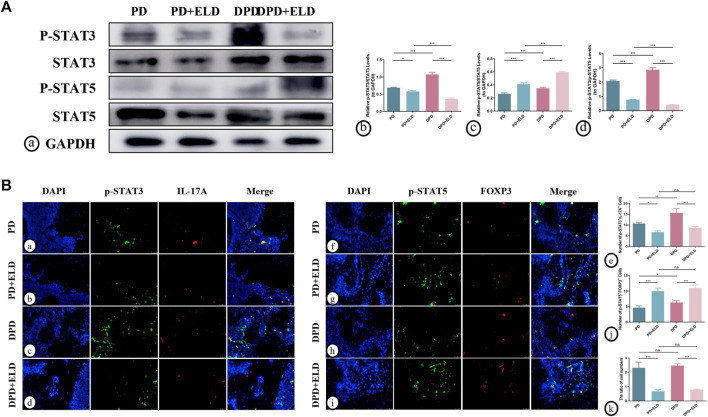
ELD regulated Th17/Treg cells through the STAT3/STAT5 signal. **(A)** (a) The protein expression of p-STAT3, STAT3, p-STAT5, STAT5 and GAPDH detected by western blotting in the PD, PD + ELD, DPD and DPD + ELD groups. The statistical analysis of (b) p-STAT3/STAT3, (c) p-STAT5/STAT5 and (d) the ratio of p-STAT3/p-STAT5 in western blotting. **(B)** The representative immunofluorescence staining images of p-STAT3^+^IL-17A^+^ cells in the (a) PD, (b) PD + ELD, (c) DPD and (d) DPD + ELD groups. (e) The statistical analysis of p-STAT3^+^IL-17A^+^ cell counts. The representative immunofluorescence staining images of p-STAT5^+^FOXP3^+^ cells in the (f) PD, (g) PD + ELD, (h) DPD and (i) DPD + ELD groups. (j) The statistical analysis of p-STAT5^+^FOXP3^+^ cell counts. (k) The ratio of p-STAT3^+^IL-17A^+^/p-STAT5^+^FOXP3^+^ cells. Data were shown as mean ± SD (*p < 0.05. **p < 0.01. ***p < 0.001).

To further clarify the molecular mechanisms underpinning ELD-induced preventative effects on Th17/Treg imbalance, western blotting and immunofluorescence were performed to evaluate STAT3/STAT5 signaling. P-STAT3 and p-STAT5 were used to detect active phosphorylated protein forms. Western blotting showed higher p-STAT5/STAT5 and lower p-STAT3/STAT3 expression levels in PD + ELD and DPD + ELD groups when compared with PD and DPD groups (Figure 7A). Statistical analyses confirmed our observations (Figure 7A), and further evaluations showed that p-STAT3/p-STAT5 expression level ratios decreased more significantly in the DPD + ELD groups (Figure 7A).

Additionally, immunofluorescence showed that p-STAT5^+^FOXP3^+^ expression was significantly enhanced in the periodontal tissue cells from PD + ELD and DPD + ELD groups when compared with PD and DPD groups. (Figure 7B). Moreover, p-STAT3^+^IL-17A^+^ expression in PD + ELD and DPD + ELD groups decreased significantly (Figure 7B). Further statistical analyses showed that the cell ratios decreased in PD + ELD and DPD + ELD groups when compared with PD and DPD groups (Figure 7B). These findings suggested that ELD exerted preventative effects on Th17/Treg cell imbalance *via* STAT3/STAT5 signaling.

## 4 Discussion

In this study, the DPD group showed significant alveolar bone loss when compared with the control group and the PD group. Moreover, a circulating and local Th17/Treg cell imbalance was observed in PD and DPD groups by detecting Th17/Treg cell ratios in the spleen and alveolar bone; in particular, the DPD group had a more serious imbalance. Surprisingly, ELD was more advantageous in preventing alveolar bone loss in the DPD group, and further investigations showed that ELD exerted preventative effects on Th17/Treg cell imbalance. Although ELD showed a good preventive effect in the DPD group, Th17/Treg cell ratios did not return to normal levels in PD + ELD and DPD + ELD groups.

In previous studies, db/db mice have been widely used to simulate diabetes (Suriano et al., 2021). In our study, ligation combined with Pg-LPS injection was used to establish an experimental periodontitis model. Micro-CT and HE staining showed obvious alveolar bone loss in PD and DPD models, indicating that our approach successfully induced periodontitis and diabetes-associated periodontitis. Compared with the control and PD group, micro-CT and histomorphological examinations showed more serious periodontal destruction in the DPD group, manifested by more alveolar bone loss and fewer bone islands. Physiologically, alveolar bone undergoes an orderly process of bone remodeling, including bone resorption and bone formation (Li et al., 2021). Periodontitis is essentially an imbalance in bone remodeling homeostasis which is induced by inflammation and immune responses, ultimately resulting in alveolar bone loss (Becerra-Ruiz et al., 2022). We identified decreased osteoblastic bone formation capacity (ALP-positive) and increased osteoclastic bone resorption capacity (TRAP-positive) in DPD group when compared with control and PD group, suggesting a more severe bone remodeling imbalance which showed less bone formation and more bone resorption. To further explore osteoclast activity, we investigated RANKL and OPG expression. It is accepted that the RANKL/RANK/OPG system has important roles in osteoclast function and bone remodeling (Tobeiha et al., 2020). RANKL increases osteoclast formation and enhances activity by interacting with RANK (homotrimeric transmembrane receptor from the tumor necrosis factor family). In contrast, OPG, a soluble RANKL decoy receptor, inhibits RANKL-RANK interactions, preventing osteoclast formation, and osteoclastic bone resorption (Udagawa et al., 2021). We found that RANKL to OPG expression ratios increased in PD and DPD mice but was more pronounced in DPD animals. Therefore, to a certain extent, changes in osteoclast activity were involved in alveolar bone loss in PD and DPD groups, which may be related to immune imbalance.

Th17 and Treg cells are specific CD4^+^ T-lymphocyte subsets with important roles maintaining immune homeostasis (Lee, 2018). In local and circulating PD and DPD group studies, we observed relatively elevated Th17/Treg ratios when compared with the control group, consistent with clinical data showing Th17/Treg cell imbalance in patients with periodontitis (Zheng et al., 2019). Furthermore, our statistical analyses showed that relative Treg cell numbers were elevated at the local microenvironment but decreased in the systemic environment. This difference may have been due to the fact that immune cells, especially T cells, are substantially elevated in local inflammation environments (Dutzan et al., 2016). In recent years, studies have indicated that Th17 and Treg cells have important roles in bone remodeling homeostasis (Cafferata et al., 2021). Th17 lymphocytes produce pro-inflammatory cytokines, such as IL-6, IL-17A, IL-23, and RANKL, to promote osteoclast differentiation (Huang et al., 2022). However, Treg cells secrete anti-inflammatory cytokines, such as IL-10 and TGF-β1 which inhibit osteoclast differentiation (Zhu et al., 2020). Hence, we hypothesize that Th17/Treg cell imbalance has important roles in PD and DPD development. We also observed that the Th17/Treg cell imbalance was more pronounced in DPD when compared with PD animals. Furthermore, it was previously reported that Th17/Treg cell imbalance was involved in insulin resistance and led to hyperglycemia (Tao et al., 2019). In turn, hyperglycemia converts Treg cells to Th1 or Th17 cells, thereby exacerbating this imbalance (Zhang et al., 2021a) and impairing defense systems in periodontal tissue *via* the release of inflammatory factors, such as IL-17, to significantly increase periodontitis risk and severity (Huang et al., 2020a). This evidence suggests that Th17/Treg cell imbalance is not only a common immune mechanism in periodontitis and diabetes, but is key to their relationship in DPD pathogenesis. Hence, based on these observations, correcting the Th17/Treg cell imbalance may be an effective treatment modality for PD and DPD, particularly DPD. Clinical and experimental data indicated that vitamin D supplementation benefited periodontal health (Han et al., 2019; Meghil et al., 2019). In our study, for the first time, we explored the preventative effects of ELD toward PD and DPD. Micro-CT and histomorphological examinations identified less alveolar bone loss in PD + ELD and DPD + ELD groups when compared with PD and DPD groups, respectively, suggesting ELD exerted preventative effects on alveolar bone loss caused by periodontitis. More interestingly, this bone loss in PD + ELD and DPD + ELD groups was broadly similar. Since alveolar bone resorption was more severe in DPD than in PD groups, and the final alveolar bone loss height was similar in both groups, our findings suggest that ELD was more advantageous in preventing DPD-induced alveolar bone loss when compared with PD. In addition, in this study, we found ELD has a hypoglycemic effect and it just presented in DPD mice but not in PD group (Supplementary Fig. 1). It was well known that impairment in glucose has an unfavorable effect on bone remodeling by the accumulation of AGEs and directly affecting the activity of osteoblast and osteoclast (Lecka-Czernik, 2017). It may be part of the reason why ELD has an advantage in preventing alveolar bone loss in DPD group.

Further, we compared the regulatory effects of ELD on Th17/Treg cell imbalance in DPD and PD groups. Our results showed that ELD did partially prevent Th17/Treg cell imbalance in PD and DPD animals, but we do not have enough evidence to prove the better preventive effect of ELD on Th17/Treg balance in the DPD group. This observation suggested that the preventive effect of ELD on DPD was not solely dependent on preventing Th17/Treg cell imbalance. Our previous studies have shown that ELD can improve the state of diabetic osteoporosis through promoting M2 macrophage polarization, which is accompanied by a decrease in blood glucose (Lu et al., 2022). Because of the immune imbalance in diabetes, the control of blood glucose is beneficial to inhibit the secretion of inflammatory factors by pro-inflammatory immune cells, promote the immunosuppressive effect of anti-inflammatory immune cells, thus indirectly regulate the balance of bone metabolism (Zhang et al., 2021b). In addition, we also found that after ELD treatment, CD4^−^ IL-17^+^ cells increased in DPD group, whereas reduced in PD group (Figures 5A, B). This may because that there are a variety of CD4^−^IL-17A^+^ cells in spleen lymphocytes, such as γδ T cells, LTi cells, some CD8^+^ T cells and B cells and so on (Gu et al., 2013), and active vitamin D may have a complex regulatory effect on immune cells under different conditions (Baeke et al., 2010). Therefore, ELD may play a role in bone remodeling through multiple pathways, and its specific mechanism needs to be further explored.

As ELD significantly improved Th17/Treg cell imbalance *in vivo*, a comprehensive exploration of its molecular mechanisms was warranted. We focused on classical STAT3/STAT5 signaling and observed that ELD not only inhibited STAT3 signaling but also activated STAT5 signaling. Additionally, p-STAT3^+^IL-17A^+^/p-STAT5^+^FOXP3^+^ cell ratios decreased significantly *via* ELD actions, indicating an association between ELD and STAT3/STAT5 signaling. From previous studies, STAT3 signaling appeared to promote Th17 differentiation, while STAT5 signaling promoted Treg differentiation, with a balance observed between signaling (Zhao et al., 2018). Thus, in PD and DPD, low STAT3 and high STAT5 activities were vital to prevent Th17/Treg cell imbalance. These findings supported our hypothesis that ELD exerted its preventative effects on PD and DPD *via* STAT3/STAT5 signaling. At present, there are few studies on how active vitamin D affects STAT3 and STAT5, but the research on JAK/STAT (Zhou et al., 2022), SOCS3/STAT (Wang et al., 2020) signaling pathway has entered the public view. Therefore, we hypothesized that ELD may regulate the phosphorylation of STAT3 and STAT5 through some regulatory factors. As latent cytoplasmic transcription factors, STATs can convey signals from the cell surface to the nucleus. This process requires the activation by cytokines and growth factors so that STATs undergo phosphorylation, involved in diverse biological functions (Abroun et al., 2015). Therefore, ELD may regulate the phosphorylation of STAT3 and STAT5 through cytokines and growth factors, and play an indirect rather than direct role, but the underling mechanism may need further exploration. In addition, though we used alveolar bone as a source of protein for western blotting, which not only contained CD4^+^T cells but also comprised other cells such as bone cells and may affect the results of western blotting. But fluorescent double staining partially can reflect the changes of STAT3 and STAT5 in Th17 and Treg cells. Identifying definitive signaling mechanisms may make us better understand ELD actions and provide new concepts for future ELD applications.

In this study, we administered ELD to the whole body *via* gavage and focused on its preventative rather than therapeutic effects toward DPD. Therefore, a study limitation may be that in an already inflamed microenvironment, high protease, low pH, and increased angiogenesis levels existed (Leblebicioglu et al., 1996; Oikonomopoulou et al., 2018; Wei et al., 2021), and therefore, altering Treg/Th17 cell ratios by applying drugs to locally induce cell differentiation may not have been as effective. Considering alveolar bone loss in DPD, which is difficult to recover from, preventing DPD may be a better approach. However, when considering alveolar bone loss, further investigations are required to determine if ELD can reverse inflammation and generate periodontal regeneration. Additionally, we observed that ELD displayed some advantages in improving DPD. Considering the status of DPD treatment, simple local basic treatments may be ineffective. Therefore, ELD has the potential to become an important systemic adjuvant drug based on immune regulation, and combined applications with local basic therapies may enhance its efficacy. However, further studies are required to address this hypothesis. We also identified some study limitations related to experimental design and our data. One study limitation was that our conclusions were mainly based on animal experiments, without *in vitro* cell validation studies. *In vivo* studies were only conducted using single-time detection, without detecting Th17/Treg changes at multiple time intervals. In future investigations, we will focus on *in vitro* studies to determine if a high glucose environment affects Th17/Treg cell imbalance and if ELD improves this high glucose-mediated imbalance.

**FIGURE 8:**
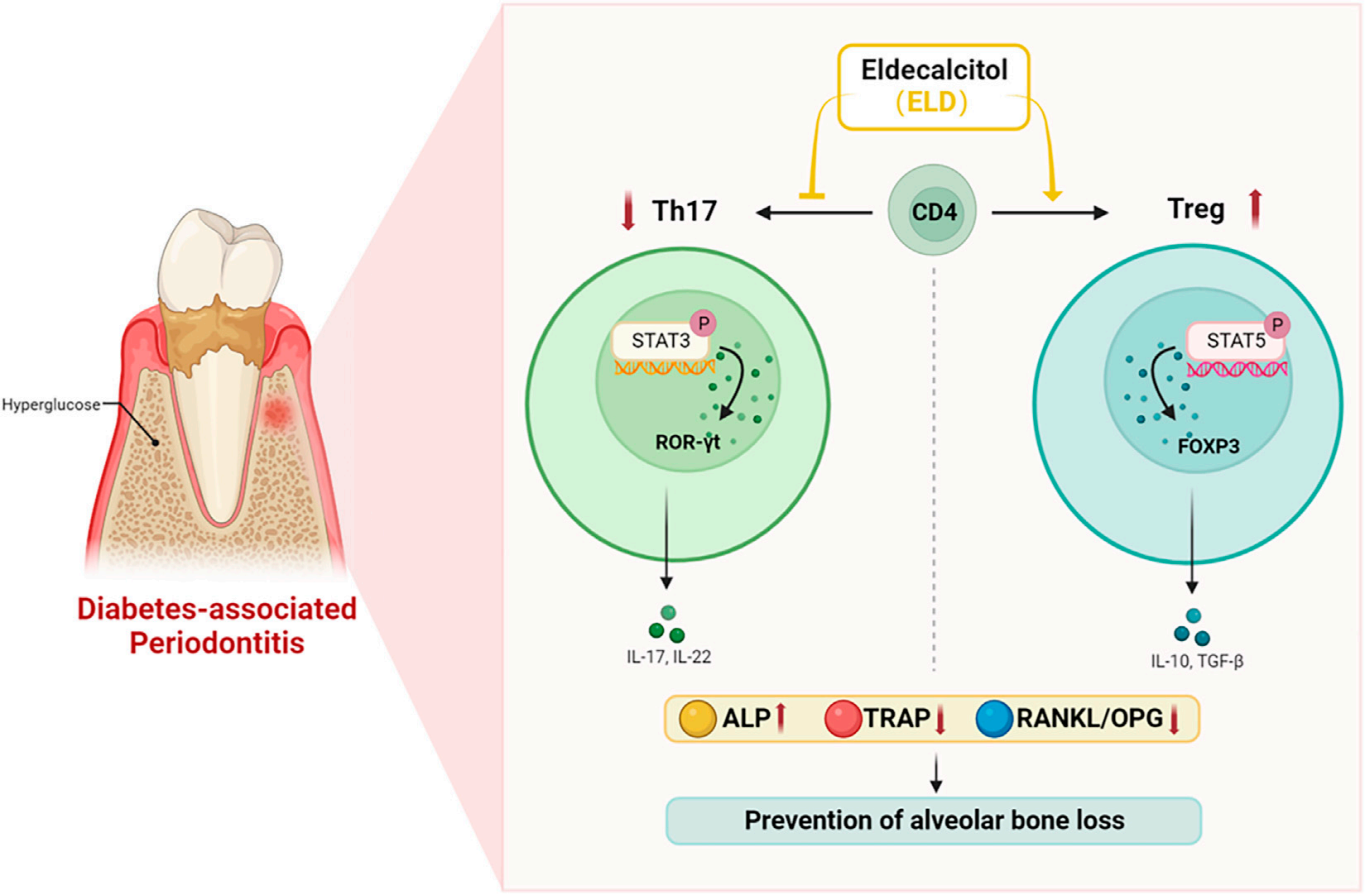
ELD effectively prevented alveolar bone loss by partially correcting the imbalance in Th17/Treg cells in DPD *via* the STAT3/STAT5 signal. ELD prevented alveolar bone loss in DPD by partially correcting the imbalance in Th17/Treg cells. One possible action mechanism may be that ELD corrected the imbalance in Th17/Treg cells by the STAT3/STAT5 signal.

In summary, ELD was more advantageous in preventing alveolar bone loss by partially correcting Th17/Treg cell imbalance in DPD. As a recently launched drug in China, ELD provides a promising adjuvant therapeutic against bone loss in DPD patients (Figure 8).

## Data Availability

The raw data supporting the conclusion of this article will be made available by the authors, without undue reservation.
