# Supplementary figures and images for: Eldecalcitol effectively prevents alveolar bone loss by partially improving Th17/Treg cell balance in diabetes-associated periodontitis
Source: Front Bioeng Biotechnol. 2023 Feb 3;11:1070117. doi: 10.3389/fbioe.2023.1070117 (PMC9936814; doi:10.3389/fbioe.2023.1070117)

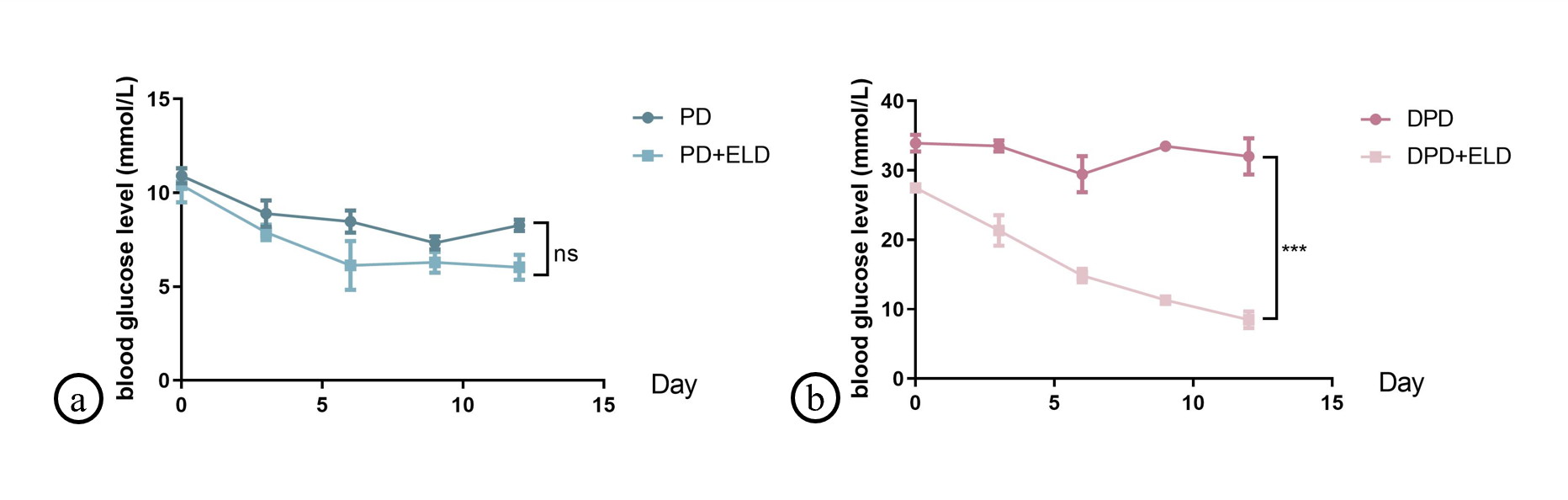

Supplement: Supplementary file 1 [file Image1.TIF]
